# *In vivo* measurement of NADH fluorescence lifetime in skeletal muscle via fiber-coupled time-correlated single photon counting

**DOI:** 10.1142/s179354582350030x

**Published:** 2024-01-30

**Authors:** Kathryn M. Priest, Jacob V. Schluns, Nathania Nischal, Colton L. Gattis, Jeffrey C. Wolchok, Timothy J. Muldoon

**Affiliations:** Department of Biomedical Engineering University of Arkansas, Fayetteville, AR, USA

**Keywords:** Glycolysis, oxidative phosphorylation, energy metabolism, volumetric muscle loss

## Abstract

Nicotinamide adenine dinucleotide (NADH) is a cofactor that serves to shuttle electrons during metabolic processes such as glycolysis, the tricarboxylic acid cycle, and oxidative phosphorylation (OXPHOS). NADH is autofluorescent, and its fluorescence lifetime can be used to infer metabolic dynamics in living cells. Fiber-coupled time-correlated single photon counting (TCSPC) equipped with an implantable needle probe can be used to measure NADH lifetime *in vivo*, enabling investigation of changing metabolic demand during muscle contraction or tissue regeneration. This study illustrates a proof of concept for point-based, minimally-invasive NADH fluorescence lifetime measurement *in vivo*. Volumetric muscle loss (VML) injuries were created in the left tibialis anterior (TA) muscle of male Sprague Dawley rats. NADH lifetime measurements were collected before, during, and after a 30 s tetanic contraction in the injured and uninjured TA muscles, which was subsequently fit to a biexponential decay model to yield a metric of NADH utilization (cytoplasmic vs protein-bound NADH, the A_1_τ_1_/A_2_τ_2_ ratio). On average, this ratio was higher during and after contraction in uninjured muscle compared to muscle at rest, suggesting higher levels of free NADH in contracting and recovering muscle, indicating increased rates of glycolysis. In injured muscle, this ratio was higher than uninjured muscle overall but decreased over time, which is consistent with current knowledge of inflammatory response to injury, suggesting tissue regeneration has occurred. These data suggest that fiber-coupled TCSPC has the potential to measure changes in NADH binding *in vivo* in a minimally invasive manner that requires further investigation.

## 1. Introduction

Cellular metabolism — critical for tissue health and development — remains a key area of biomedical research.^1–8^ Metabolic pathways, such as glycolysis and oxidative phosphorylation (OXPHOS), serve to synthesize energy from glucose into the useable form, adenosine triphosphate (ATP).^1,5,9–16^ Cellular metabolism is highly sensitive to the tissue microenvironment, which causes changes in the rates of glycolysis and OXPHOS. Perturbations in metabolism within tissue can be due to several causes such as cancer, infection, injury, and other changes in energy demand.^2,17–23^ Due to the large role metabolism plays in tissue microenvironments, the study of these perturbations has become a focus for preclinical and clinical research to understand normal and disease physiology, such as mitochondrial diseases, cancer, and diabetes.^1,3,8,24^ This may be accomplished using optical techniques for measuring the autofluorescence of metabolic cofactors, such as reduced nicotinamide adenine dinucleotide (NADH) and flavin adenine dinucleotide (FAD). This is one of many methods for measuring the fluorescence intensity and fluorescence lifetime. Other methods include multiphoton microscopy and fluorescence lifetime imaging (FLIM). The use of fluorescence intensity and/or fluorescence lifetime measurements has greatly improved our understanding of metabolism *in vitro* and, in a limited manner, *in vivo*.^3,17,19,21–23,25,26^

*In vivo* measurement of NADH fluorescence lifetime is challenging due to the significant scattering and absorption within the UV and blue spectral regions in intact tissue structures. Due to this, FLIM measurements are typically limited to superficial tissues or may be highly invasive requiring surgical intervention to expose the tissue of interest.^24,27^ This presents a significant challenge in metabolic research when performing NADH fluorescence measurements in longitudinal small animal models.^17,24,27–29^ Fiber-coupled time-correlated single photon counting (TCSPC) could help address these limitations by providing a minimally invasive method for measurement of NADH fluorescence lifetime. This method uses a multimode optical fiber fit with an implantable needle “optrode“ for point-based measurement of NADH fluorescence lifetime.^27^ A comparison of fiber-coupled TCSPC and FLIM can be found in Supplemental Table 1. In this study, fiber-coupled TCSPC was used to measure the changes in NADH protein-binding in skeletal muscle.

In the cytoplasm, glycolysis breaks down glucose in a series of ten chemical reactions resulting in pyruvate, ATP and NADH, a metabolic cofactor that holds a prominent role in cellular metabolism as an electron/proton carrier.^2,5,9,13–16,28,30^ The NADH and pyruvate that were generated from glycolysis are then used in the tricarboxylic acid (TCA) cycle and the electron transport chain (ETC) within the mitochondria during OXPHOS.^31^ NADH binds to proteins within the electron transport chain to aid in the generation of ATP and water.^4,9,32^ The rates of these reactions often change because of changes within the tissue microenvironment and can be measured by the ratio of free to protein-bound NADH. This ratio serves as a surrogate marker of the relative rates of glycolysis, where NADH remains in a free or soluble form, and oxidative phosphorylation, where NADH binds to a protein in the ETC. Changes in the rates of these metabolic processes in relation to each other can provide insight into how ATP generation changes in response to different stimuli or changes within the tissue.^2,28,45,46^ This study served to measure NADH fluorescence lifetime in skeletal muscle tissue during times of high and low energy demand, such as muscular contraction and post-injury tissue repair. To do this, NADH lifetime was compared in uninjured and injured muscles in a rat model before, during, and after tetanic contraction. We hypothesized that fiber-coupled TCSPC would detect changes in NADH protein binding within muscle tissue in these metabolically dynamic conditions. To our knowledge, this study is the first to utilize this approach to measure NADH fluorescence lifetime during and immediately following muscular contraction.

## 2. Materials and Methods

### 2.1. Animal model

Twelve-week-old male Sprague Dawley rats (*n* = 4), weighing between 300 g and 325 g, were purchased from Envigo (Dublin, VA) for this study. The animals were housed individually at 23°C±1°C and 50 ± 10% humidity with a 12:12-h light-dark cycle and had access to water and standard rodent food ad libitum and received hydrogel (Clear H_2_O) every day. Upon arrival, the animals were allowed to acclimate for seven days prior to surgery. All animal experiments were approved by the University of Arkansas Institutional Animal Care and Use Committee (IACUC #21006) and carried out in accordance with the National Institutes of Health Guide for the Care and Use of Laboratory Animals and ARRIVE guidelines. All efforts were made to minimize animal suffering and to reduce the number of animals used.

All procedures in this study were completed using continuous inhaled isoflurane induced at 4% isoflurane in oxygen and maintained at 2–2.5% isoflurane in oxygen. Animal temperature was continuously monitored using a temperature-controlled heating pad with a rectal probe (Harvard Apparatus) and eye ointment (GenTeal) was applied to the eyes for protection from drying.

### 2.2. Volumetric muscle loss injury

VML injuries were created surgically in the left tibialis anterior (TA) muscle of each rat with the right leg serving as an internal control. The surgical site was shaved and cleansed using a povidone-iodine scrub prior to creating a 1–2 cm incision parallel to the tibia. The skin and fascia were pulled back to expose the TA muscle. An 8-mm biopsy punch (VWR) was used to create a 3 mm deep partial thickness VML defect in the center of the TA muscle. The biopsied muscle was weighed and additional material was removed to achieve a 20% of muscle mass VML. 5–0 absorbable sutures (Vicryl, Ethicon, Summerville, MA) were used to close the fascia and skin with interrupted stitches.^33–35^ Finally, a subcutaneous injection of extended-release buprenorphine (ZooPharm), at a dosage of 0.05 mg/kg, was given immediately post-surgery. Additionally, animals received a 5 mg tablet containing 2 mg of Rimadyl (BioServe) in their cage each day post-surgery for additional pain management.^36^

### 2.3. In vivo NADH fluorescence lifetime measurements

A fiber-coupled TCSPC system was obtained from Becker-Hickl. This system was composed of a TCSPC module (SPC-130-EMN), a cooled picosecond diode laser with a wavelength of 375 (+5= − 10) nm (BDS-SM-375-FBC-101), a hybrid GaAsP photodetector for photon counting (HPM-40–100), a detector control card (DCC-100), a hybrid fiber probe (F200-Hybrid-FC-COLL-OPTC), and a biconvex lens (*f* = 20 mm, *d* = 22.4 mm, O-LBK-F20-D22.4) with a 450–700 nm AR coating on both sides, which was housed in front of the detector. System accuracy was measured using an aqueous solution of unbound NADH, which exists in two molecular conformations. Previous studies have reported lifetime components of ~0.3–0.4 ns and ~0.7 ns for the two conformations, or a mean lifetime of ~0.4 ns. Our measured lifetime values of 0.405 ns and 0.754 ns (mean lifetime of 0.445 ns) were in agreement with these previous studies.^37,38^

**Fig. 1.**
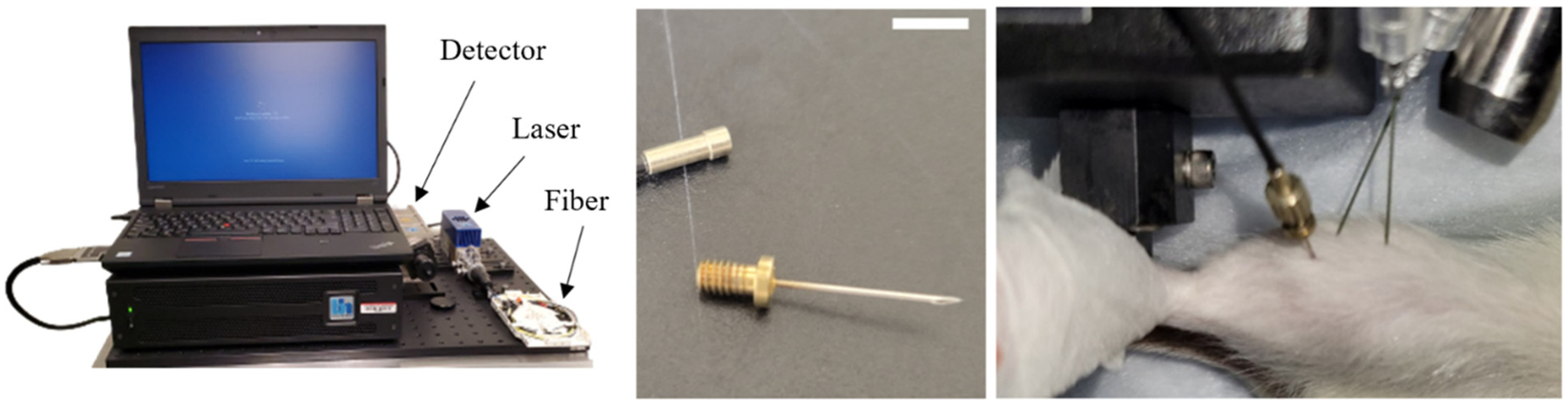
Fiber-coupled TCSPC System. Left: Image of the components of the TCSPC system. Center: Image of the removable needle optrode. Right: The TCSPC needle optrode (left) and the electrophysiology electrodes (right) inserted in the TA muscle for stimulation of contraction and measurement of NADH lifetime. Scale bar is 5 mm

A 12 mm, 26-gauge implantable needle optrode (Becker-Hickl, AP11275), shown in Fig. 1, was attached to the end of the fiber. Prior to implantation, the needle was cleaned using lens cleaning tissue (ThorLabs Inc) saturated with methanol. The needle was then inserted into the center of the TA muscle at approximately a 45−° angle. The laser was briefly turned on to confirm an adequate signal. If necessary, minor adjustments to the needle position in the muscle were made until the peak of the decay curve had a value greater than 80 photons. Data were collected using the continuous flow mode to create a time series with an integration time of 0.2 s (see Supplemental Table 2 for further information on data collection settings). Comprehensive system testing and characterization have been performed by us (see Supplemental Information) and others.^27,39,40^

NADH fluorescence lifetime and contractile torque data were collected beginning two days post-surgery. Data were collected for 30 s prior to a 30 s tetanic contraction period and continued for an additional 30 s following termination of the contraction. This was performed every other day in uninjured muscle from day zero until the endpoint was reached. Injured muscle was measured only at the endpoint.

### 2.4. Tetanic contraction

A dual-model muscle lever system (Aurora Scientific, Ontario, Canada) was used to measure contractile torque upon muscle stimulation. Tape was used to secure the foot to the lever arm and the animal was positioned with 90−° angles at the knee and ankle. The knee was stabilized to ensure no movement during stimulation. Percutaneous needle electrodes were inserted into the anterior compartment of the TA for stimulation of the peroneal nerve.^33,34^ Instant stimulation was used to optimize the current for each rat and to verify proper stimulation following the introduction of the needle optrode from the TCSPC fiber. A stimulation with a frequency of 50 Hz, a pulse width of 1 ms, and a potential of 2–5 V was induced for 30 s.^41^ A 30 s delay was programmed into the function to ensure accurate timing between the NADH lifetime measurements and the contraction following a simultaneous start. The torque of the contraction was measured by the computer-controlled servomotor attached to the foot pedal and the data were collected using commercial muscle physiology software (Aurora Scientific, Ontario, Canada). Tetanic contraction was induced every other day on the uninjured leg and at the endpoint for the injured leg. The peak torque (N-cm) was collected and normalized to the body weight of the animal (N-cm/Kg).

### 2.5. Tissue harvest and histology

Following completion of contractile torque and NADH lifetime data, the TA and extensor digitorum longus muscles from each leg were resected, washed in sterile phosphate buffered serum (PBS), and weighed on either day 6 or day 14.^34^ Excess moisture was removed from the muscle by saturation with baby powder and then the TA muscle was flash-frozen. The muscle was submerged in isopentane, and cooled in liquid nitrogen to −90°C for 10 s. Frozen tissue was placed on dry ice until they were placed into a −80°C freezer for storage. All animals were then euthanized by carbon dioxide inhalation in accordance with guidelines provided by the AVMA Panel on Euthanasia of Animals.

TA muscles were partially embedded in optimal cutting temperature compound (Tissue-Tek) and sectioned at a temperature maintained between −25°C and −20°C using a cryostat (Leica BioSystems). Tissue cross sections were obtained at a thickness of 8 *μ*m and mounted on slides for hematoxylin and eosin staining and imaging via wide-field microscopy.^33,35^ Images were analyzed in Fiji (ImageJ) via color deconvolution to estimate the areas of hematoxylin and eosin stain for uninjured and injured muscle at days 6 and 14 post-injury.^42,43^

### 2.6. NADH fluorescent lifetime data analysis

NADH fluorescent lifetime data were analyzed using SPCImage (Becker & Hickl). Upon loading in the first dataset, the instrument response function (IRF) was extrapolated from the data and saved for analysis of all remaining lifetime data.^44,45^ NADH lifetime decay curves were fitted with a biexponential decay model, shown in Eq. (1), via iterative deconvolution of the measured decay curve and the IRF using maximum-likelihood estimation (see Supplemental Table 3 for SPCImage software settings).


(1) $$f(t)=A_{1}e^{\frac{-t}{\tau_{1}}}+A_{2}e^{\frac{-t}{\tau_{2}}}.$$

The chi-squared value was calculated for each fit and ranged between 0.96 and 1.4. The contribution values (*A*_1_ and *A*_2_, where the sum of *A*_1_ and *A*_2_ is 1) and lifetime values (τ_1_ and τ_2_ were exported for plotting. The ratio of free NADH and protein-bound NADH (*A*_1_τ_1_/*A*_2_τ_2_) was plotted versus time to identify longitudinal changes.

### 2.7. Fractal dimension analysis

The three regions (pre-contraction, contraction, and post-contraction) were separated, and each underwent fractal dimension analysis. Fractal dimension analysis is a technique that can be used to detect self-similarities within shapes or repeated patterns within signals.^46–48^ This technique has been used to detect changes in signal complexity of several biological systems such as cardiovascular or neurological signals. Studying the fractal nature of biological systems can lead to a greater understanding of how the system works and how it may be affected by disease or injury.^46,47^

A Fourier transformation was applied to each contractile region and a power density spectrum was plotted.^46–48^ Next, the logarithm of the power density spectrum was plotted versus the log of the frequency. A line was fit to each log-log plot, with *R*^2^ values ranging between 0.1136 and 0.8958 (see Supplemental Table 4 for more specific details), and the slope of this line was extracted. The absolute value of the slope, *β*, was used to calculate the fractal dimension using the equation for a random walk.


(2) $$FD=\frac{5-\beta }{2}.$$

### 2.8. Statistical analysis

Fluorescence lifetime from uninjured muscle tissue was statistically analyzed using a repeated measures analysis of variance (ANOVA), while all other data were compared using a one-way ANOVA. *Post-hoc* tests, including Tukey HSD and student t-tests, were used to perform multiple comparisons. All error values presented represent the standard error of the mean (SEM).

## 3. Results

### 3.1. NADH fluorescence lifetime

#### 3.1.1. Effects of tetanic contraction on the NADH utilization in uninjured muscle tissue

**Fig. 2.**
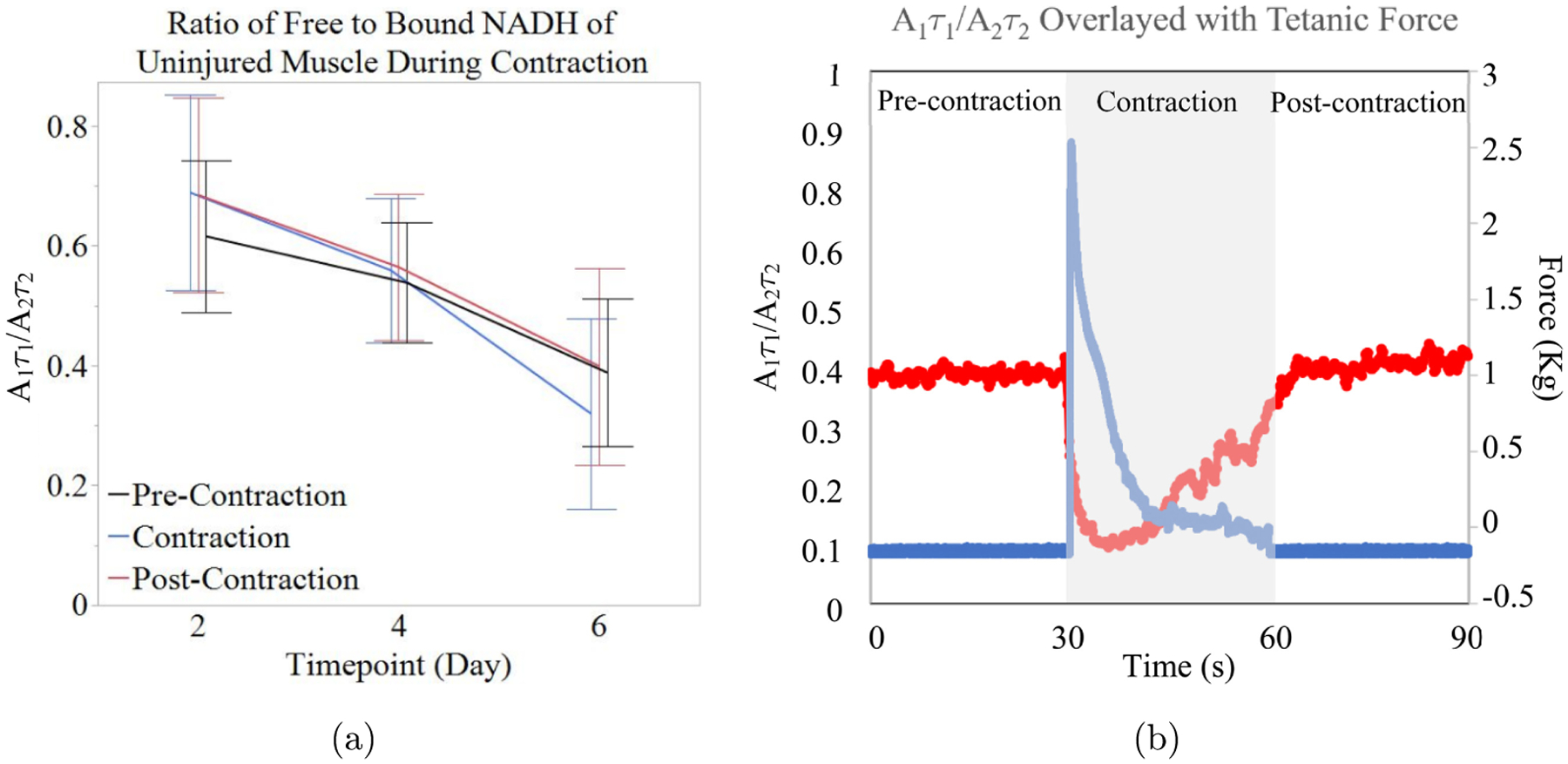
(Color online) Average ratio of free and bound NADH in uninjured muscle (a) *A*_1_τ_1_/*A*_2_τ_2_ ratio before, during, and after contraction (*n* = 4). (b) Representative single dataset showing the *A*_1_τ_1_/*A*_2_τ_2_ ratio (red) and the contractile force (blue) from one measurement. Error bars indicate the (SEM).

The average *A*_1_τ_1_/*A*_2_τ_2_ ratios for each phase of contraction were statistically compared over three days using repeated measures one-way ANOVA. There were no significant differences in the ratio of free to protein-bound NADH in muscle pre-contraction, during contraction, and post-contraction. These data did indicate a trend in the *A*_1_τ_1_/*A*_2_τ_2_ ratio, showing an increase during (0.689 ± 0.164, 0.559 ± 0.121, and 0.319 ± 0.159, for each day, respectively) and after (0.695 ± 0.162, 0.564 ± 0.122, and 0.399 ± 0.164, for each day, respectively) contraction compared to pre-contraction (0.616 ± 0.126, 0.538 ± 0.101, and 0.388 ± 0.123, for each day, respectively); however, on day 6 there was a decrease in this ratio during the contraction, which can be seen in Fig. 2(a).

To visualize these changes in NADH binding, Fig. 2(b) presents data from one continuous measurement. In this figure, an average *A*_1_τ_1_/*A*_2_τ_2_ ratio of approximately 0.429 ± 0.001 can be seen for the 30 s measurement prior to muscular contraction. Upon stimulation of the tetanic contraction, the ratio decreased to an average of 0.204 ± 0.006 for the 30 s contraction. After the muscle returned to rest, the *A*_1_τ_1_/*A*_2_τ_2_ ratio increased to 0.443 ± 0.002, which is slightly higher than it was before contraction.

#### 3.1.2. Effects of a volumetric muscle loss injury on NADH binding during tetanic contraction

**Fig. 3.**
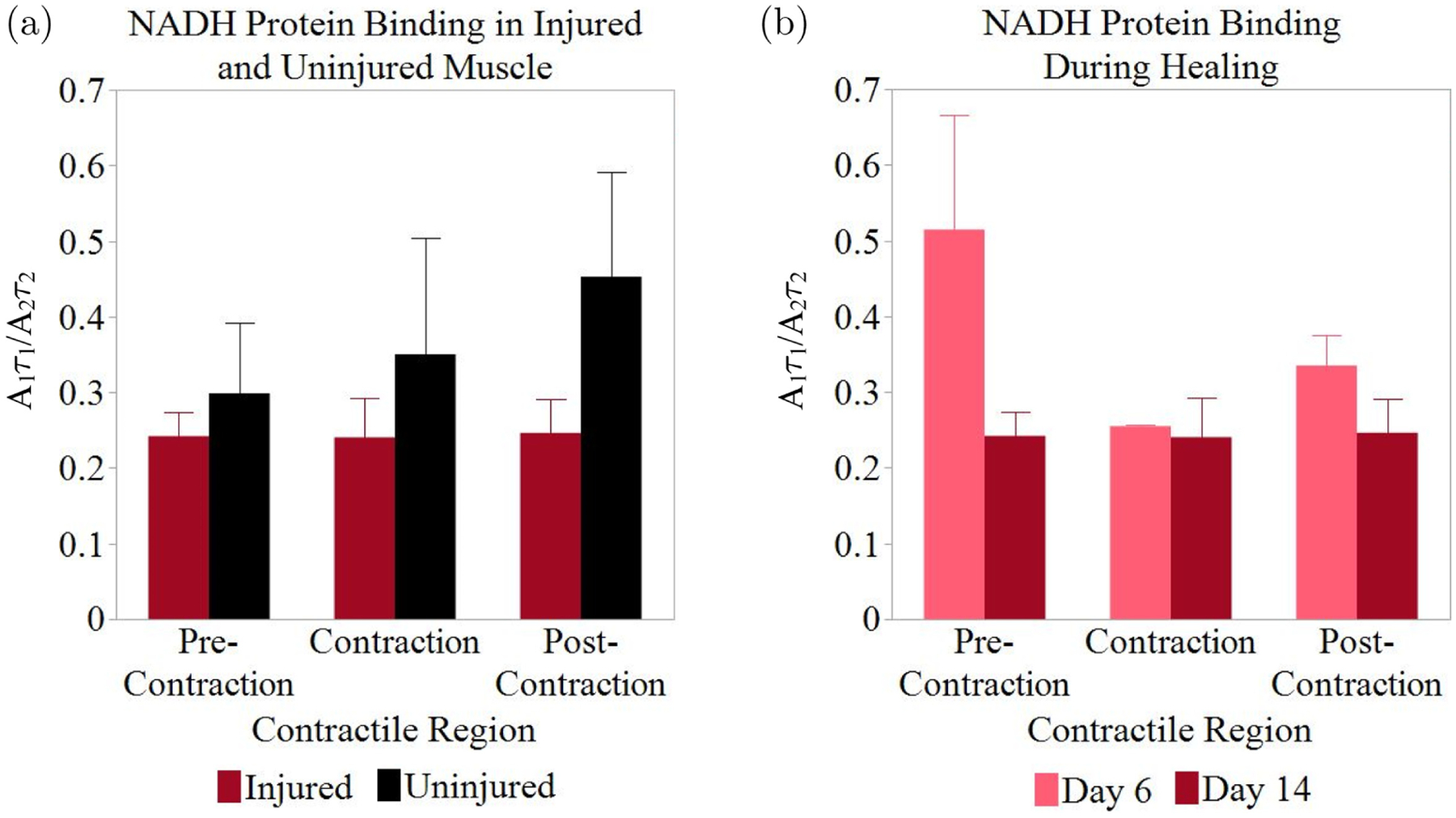
Comparison of NADH utilization (a) in injured and uninjured muscle on day 14 (*n* = 2) and (b) 6- and 14-days post-injury. Error bars indicate SEM.

NADH fluorescence lifetime in uninjured muscle tissue was then compared with that of injured muscle tissue. Measurements were repeated in VML injuries to compare NADH binding in uninjured tissue with tissue undergoing an inflammatory response for tissue repair. The uninjured muscle was compared to injured muscles 14 days post-injury. Figure 3(a) shows a comparison of the ratio of free to protein-bound NADH in the muscle tissue in each of the three phases of contraction. There were no statistically significant differences between uninjured and injured muscle, but the trend in the changes of the *A*_1_τ_1_/*A*_2_τ_2_ ratio appears to change in injured muscle. While uninjured muscle displayed a low *A*_1_τ_1_/*A*_2_τ_2_ ratio before contraction (0.299 ± 0.093) with an increase upon stimulation of tetanus (0.350 ± 0.153) and following contraction (0.453 ± 0.139), the *A*_1_τ_1_/*A*_2_τ_2_ ratio in injured muscle remained relatively consistent across all regions (0.242 ± 0.032, 0.240 ± 0.053, and 0.247 ± 0.045, respectively).

#### 3.1.3. Effects of muscle tissue healing on NADH binding in relation to tetanic contraction

The effects of muscle injury on NADH lifetime were further investigated by comparing injured muscle at day 6 and day 14 post-injury during these three phases of contraction. Figure 3(b) shows the *A*_1_τ_1_/*A*_2_τ_2_ ratio of the three phases at these two time points. Day 6 (*n* = 2) indicated a greater variability in the *A*_1_τ_1_/*A*_2_τ_2_ ratio between the three regions, though it was not statistically significant. On day 6, the *A*_1_τ_1_/*A*_2_τ_2_ ratio was very high prior to contraction at 0.515 ± 0.152 but dropped to 0.255 ± 0.002 upon stimulation. At the end of the 30 s contraction, the ratio increased to 0.335 ±0.040. On day 14 (*n* = 2), injured muscle displayed very minimal changes in the *A*_1_τ_1_/*A*_2_τ_2_ ratio from one phase of contraction to the next (0.242 ± 0.032, 0.240 ± 0.053, 0.247 ± 0.045 for pre-contraction, contraction, and post-contraction, respectively).

### 3.2. Fractal dimension

#### 3.2.1. Effects of tetanic contraction on the fractal dimension of NADH utilization in uninjured muscle tissue

**Fig. 4.**
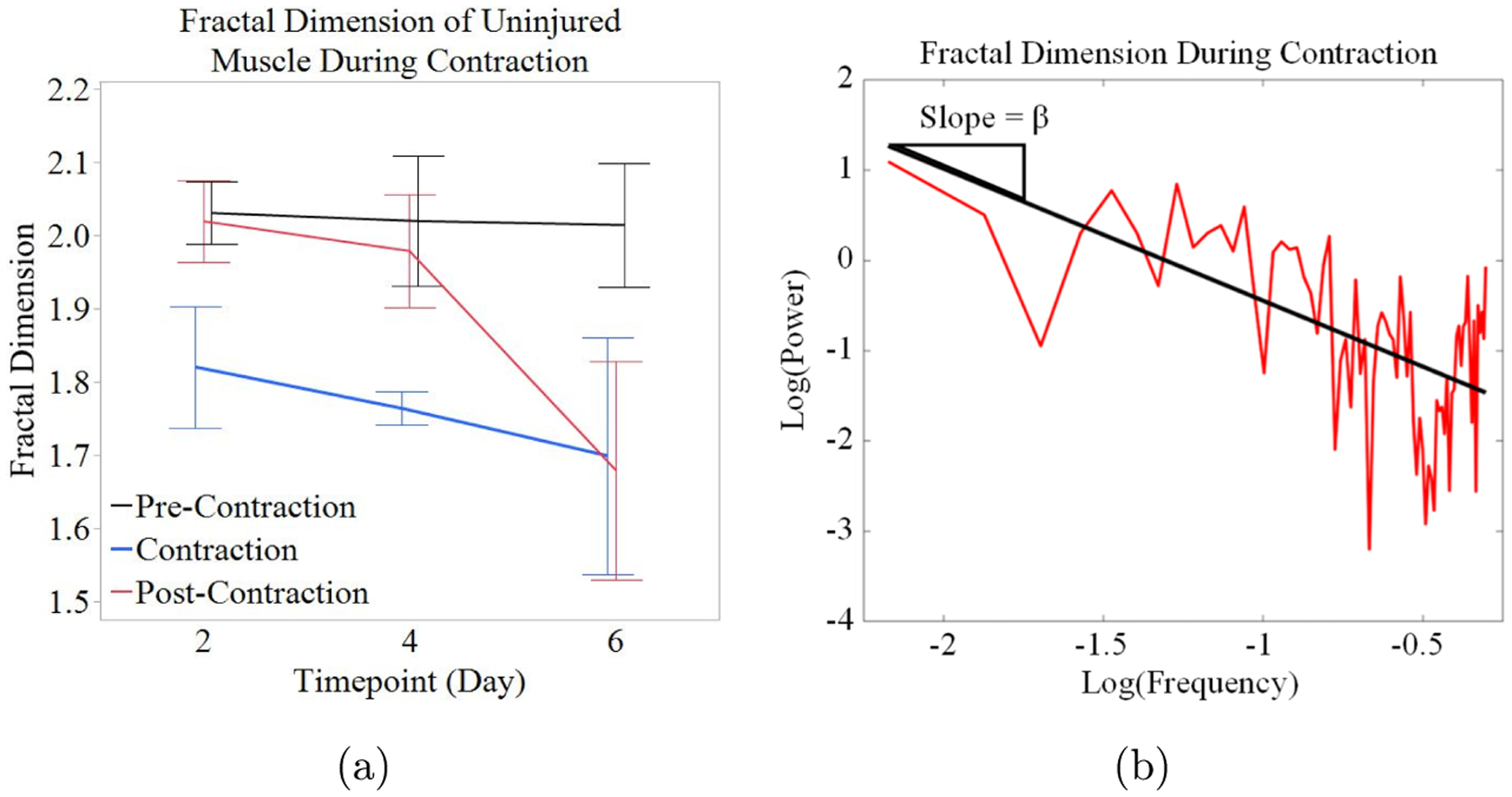
(Color online) Fractal dimension of the average ratio of free and bound NADH in uninjured muscle (a) Average fractal dimension of the *A*_1_τ_1_/*A*_2_τ_2_ ratio before, during, and after contraction (*n* = 4). (b) Representative plot of fractal dimension calculation. Transformed data (red) and fit line (black). Error bars indicate the (SEM).

The *A*_1_τ_1_/*A*_2_τ_2_ ratios were further analyzed via fractal dimension analysis by using the power spectral density method.^46–48^ The fractal dimension of each region, pre-contraction, contraction, and post-contraction, was calculated. Figure 4(a) shows the average fractal dimensions of the phases of contraction. There were no significant differences in the fractal dimension values measured, but the data indicate a trend. The fractal dimension is consistently higher prior to contraction (2.030 ± 0.043, 2.019 ± 0.089, and 2.014 ± 0.084, for each day, respectively) than it is during (1.820 ± 0.083, 1.764 ± 0.023, and 1.699 ± 0.161, for each day, respectively) and after the contraction (2.019 ± 0.056, 1.978 ± 0.077, and 1.679 ± 0.149, for each day, respectively). On days 2 and 4, there was a decrease in the fractal dimension during the contraction, which then increased again post-contraction. Day 6 shows similar trends, but the fractal dimension was lowest post-contraction. Figure 4(b) provides an example of the fractal dimension calculation via the power spectral density method for the contraction region of one measurement.

#### 3.2.2. Effects of a volumetric muscle loss injury on the fractal dimension of NADH utilization during tetanic contraction

**Fig. 5.**
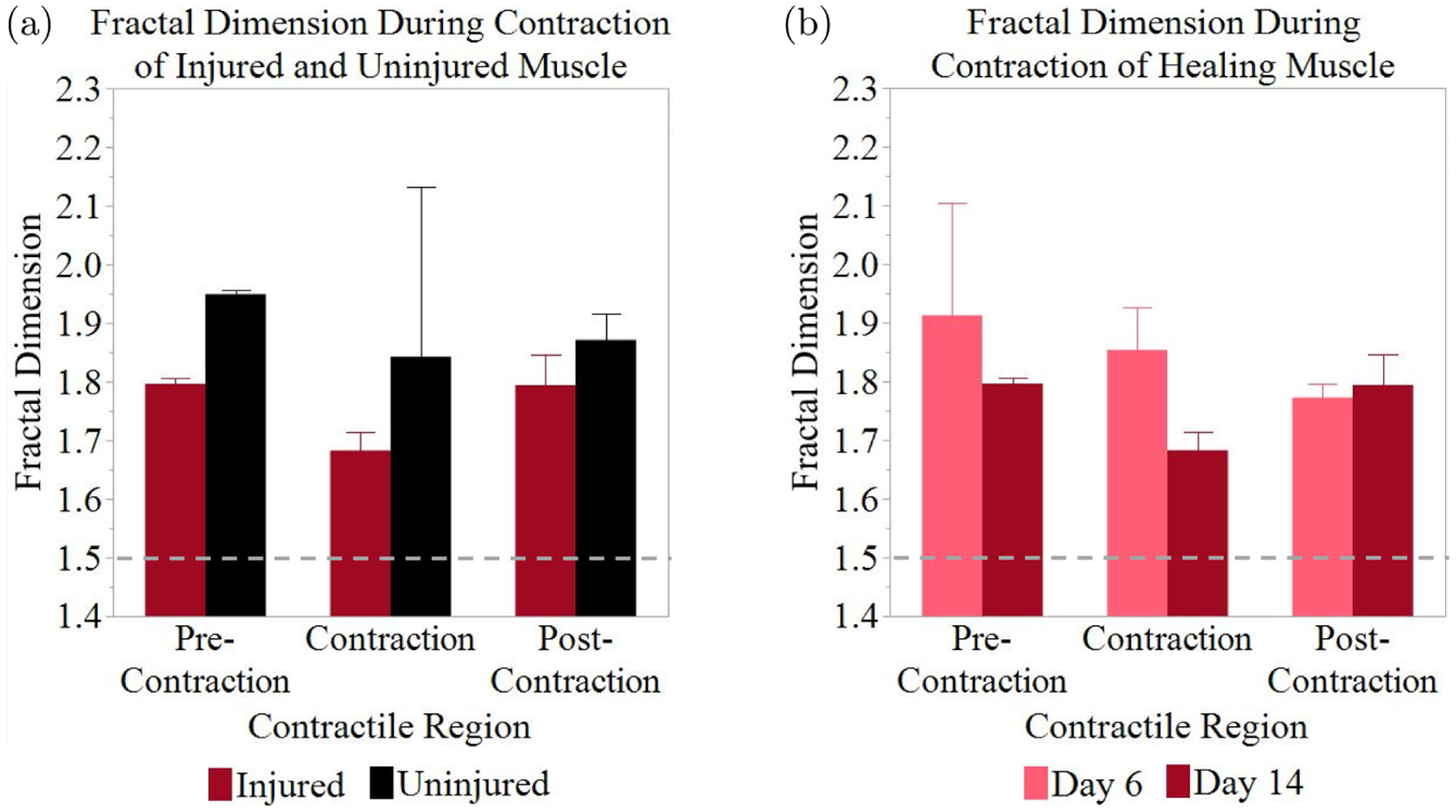
(Color online) Comparison of the fractal dimensions of (a) injured and uninjured muscle on day 14 (*n* = 2) and (b) muscle 6- and 14-days post-injury (*n* = 2). Dashed line indicates the value for a random walk. Error bars indicate SEM.

The fractal dimension, shown in Fig. 5(a), displays very similar trends between uninjured and injured muscles. The fractal dimension remains higher in the uninjured muscle tissue across all three phases of contraction although no significant difference was observed. Uninjured muscle displayed a slight decrease in the fractal dimension during contraction which increased again post-contraction (1.949 ± 0.007, 1.843 ± 0.289, and 1.871 ± 0.046, respectively). The same trend can be seen in injured muscle (1.796 ± 0.010, 1.683 ± 0.032, 1.794 ± 0.052, respectively).

The fractal dimension (Fig. 5(b)) displayed no significant differences but did show a change in the trends between day 6 and day 14. On day 6, the fractal dimension was very high pre-contraction (1.913 ± 0.190), then dropped considerably during contraction (1.854 ± 0.072) then decreased again post-contraction (1.773 ± 0.024). On day 14, the fractal dimension was low pre-contraction (1.796 ± 0.010), decreased during contraction (1.683 ± 0.032), and increased again post-contraction (1.794 ± 0.052). All fractal dimension values remained above the random walk threshold of 1.5.

### 3.3. Histology

#### 3.3.1 Structural and functional measurements of injured and uninjured muscle

**Fig. 6.**
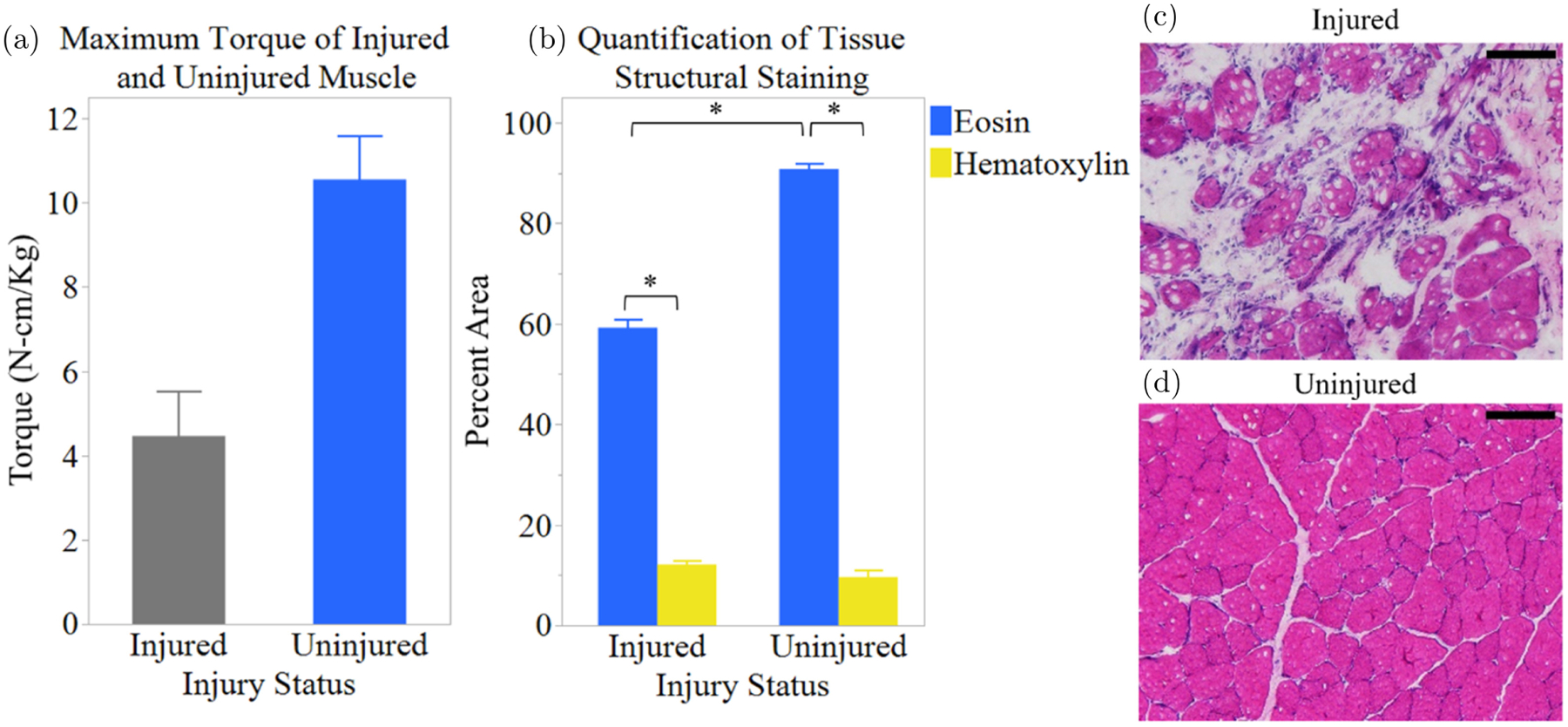
(Color online) Comparison of injured and uninjured muscle on day 14 post-injury (*n* = 2). (a) Maximum torque. (b) Tissue structure ((c)–(d)) H&E image of (c) injured and (d) uninjured muscle. Error bars indicate SEM. Scale bars are 100 *μ*m.*

Maximum torque measurements and histology (Fig. 6) were used to quantify differences in the functional and structural difference in the uninjured and injured muscle on day 14 to compare with reports in the literature. Injured muscle displayed an average maximum torque of 4.460 ± 1.072 N-cm/Kg, which was less than half that of the uninjured muscle tissue at 10.548 ± 1.044 N-cm/Kg (Fig. 6(a)). These results are comparable to previously published VML studies which reported the maximum torque output as approximately 50% lower in TA muscles following VML injury when compared to uninjured TA muscle.^33,34^

Muscle tissue cross-sections were stained with hematoxylin and eosin to visualize the overall cellularity of the tissue. There was a statistically significant (*p* < 0.0001) difference in the structural components of uninjured and injured muscle tissue (Fig. 6(b)). In uninjured muscle tissue, the hematoxylin-stained nuclei accounted for approximately 9.602 ± 1.310% and the eosin-stained extracellular matrix, cytoplasm, and other structures accounted for 90.758 ± 1.145% of the tissue by area. Injured muscle, on the other hand, displayed approximately 12.117 ± 0.670% nuclei and 59.219 ± 1.731% other structures, such as cytoplasm and extracellular matrix. Figures 6(c) and 6(d) show images of uninjured and injured muscle tissue stained with H&E at day 14 post-injury. Uninjured muscle tissue displayed highly organized muscle cells, while injured muscle showed a greater area of tissue lacking uninjured muscle cells. This consistency seen for both functional and structural results, implies that the lifetime values measured *in vivo* via fiber-based TCSPC are from typical uninjured and VML-injured muscle tissue.^33–36^

#### 3.3.2. Structural and functional measurements of healing muscle

**Fig. 7.**
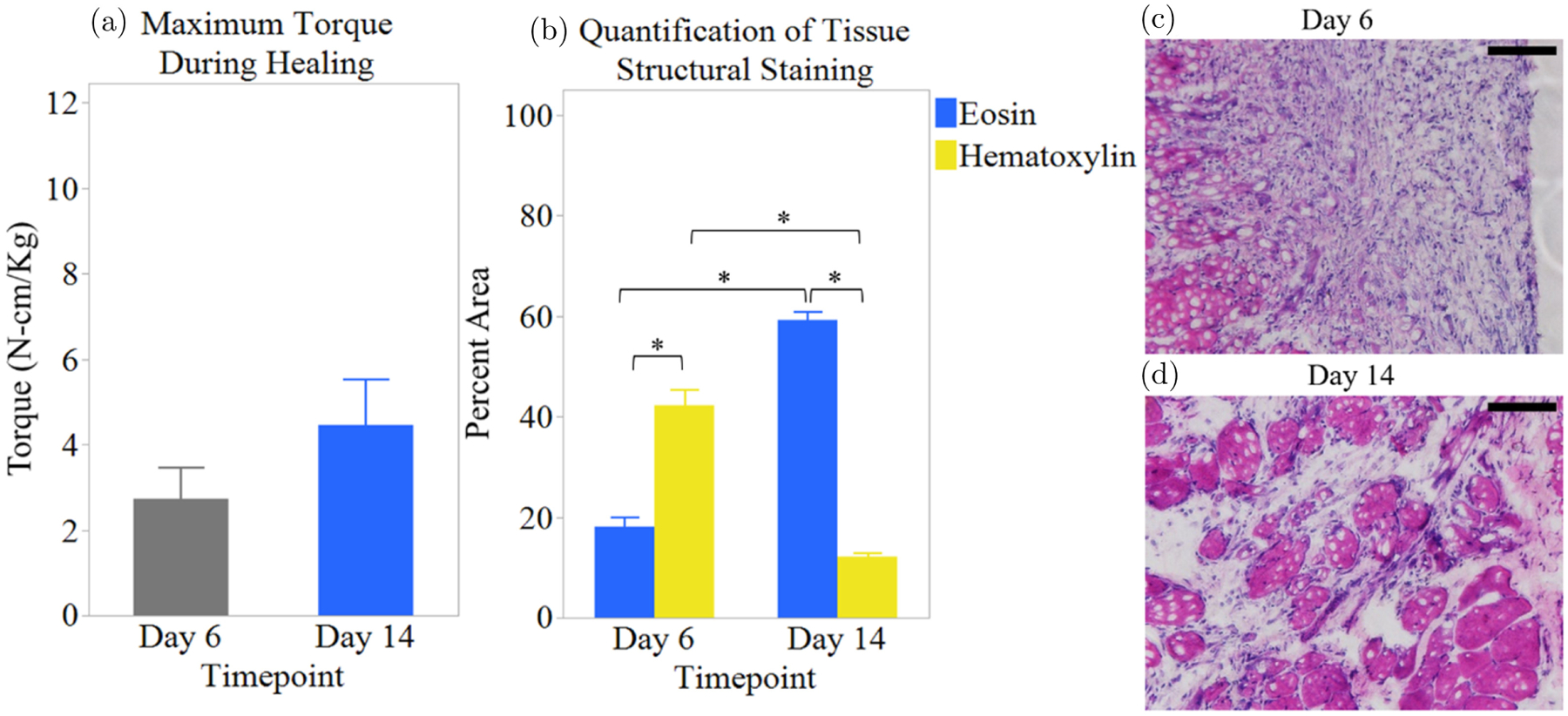
(Color online) Comparison of muscle 6- and 14-days post-injury (*n* = 2). (a) Maximum torque. (b) Tissue structure ((c)–(d)) H&E image of (c) day 6 and (d) day 14 muscle. Error bars indicate SEM. Scale bars are 100 *μ*m. * represents *p* < 0.0001.

Maximum torque measurements and histology (Fig. 7) were used to quantify functional and structural differences at the two timepoints. On day 6, injured muscles displayed an average maximum torque of 2.728 ± 0.732 N-cm/Kg. Then on day 14, the average max torque increased to 4.460 ± 1.072 N-cm/Kg (Fig. 7(a)). Muscle tissue cross-sections were stained with hematoxylin and eosin to visualize the overall cellularity of the tissue. There was a statistically significant (*p* < 0.0001) difference in the structural components of injured muscle tissue on days 6 and 14. On day 6, the hematoxylin-stained nuclei accounted for approximately 42.235 ± 3.133% and the eosin-stained extracellular matrix and cytoplasm accounted for 18.102 ± 1.868% of the tissue by area. On day 14, there was approximately 12.117 ± 0.670% nuclei and 59.219 ± 1.731% extracellular matrix and cytoplasm. Figures 7(c) and 7(d) show images of injured muscle tissue stained with H&E at days 6 and 14 post-injury. These results suggest that as the tissue had time to heal, there were increases in connective tissue and uninjured muscle cells, but they still were not comparable to the uninjured tissue. These results are consistent with previously published VML studies.^33,34^

## 4. Discussion

In this study, NADH fluorescence lifetime measured *in vivo* was influenced by a 30-s tetanic contraction. NADH utilization is presented as the ratio of free NADH (*A*_1_τ_1_) to protein-bound NADH (*A*_2_τ_2_), also referred to as the *A*_1_τ_1_/*A*_2_τ_2_ ratio. NADH is in a free or soluble form during glycolysis, but it binds to a protein in the ETC during oxidative phosphorylation. The comparison of the amount of free NADH relative to protein-bound NADH can suggest the relative rates of these reactions for ATP production.^2,28,49,50^

There were no significant differences found in the NADH lifetime data collected, but a trend emerged showing an increase in the *A*_1_τ_1_/*A*_2_τ_2_ ratio upon stimulation of tetanic contraction indicating a greater amount of free NADH (glycolysis) relative to protein-bound NADH (ETC). This could suggest that the cells are increasing their rate of glycolysis to maintain adequate levels of ATP during contraction.^2,49,50^ On average, there was a slightly higher *A*_1_τ_1_/*A*_2_τ_2_ ratio following the termination of muscular contraction compared to before contraction, but there was considerable variation between measurements. These trends suggest a possible metabolic shift in favor of glycolysis for ATP generation post-contraction.^2,49–51^

Overall, the *A*_1_τ_1_/*A*_2_τ_2_ ratio was higher (though not statistically significant) in uninjured muscle when compared to injured muscle tissue 14 days post-injury. This indicates that there is a greater amount of protein-bound NADH in injured muscle and, therefore, higher rates of oxidative phosphorylation. These results suggest that higher rates of oxidative phosphorylation, which is not consistent with the glycolytic increase that is a hallmark of the highly proliferative cells that make up the inflammatory response that is responsible for muscle repair.^2,49–51,53,54^ Future studies should incorporate additional histological staining of key markers of the inflammatory response and muscle proteomics, such as glycolytic and oxidative metabolites, to validate this increase in oxidative phosphorylation.^55–57^

Injured muscle showed very little variation in the ratio of free to protein-bound NADH across the three phases of contraction, while uninjured muscle showed increases in the *A*_1_τ_1_/*A*_2_τ_2_ ratio upon stimulation of contraction. This could be a result of the many changes to the tissue microenvironment throughout the process of healing from the early pro-inflammatory step to the pro-inflammatory response and finally the restoration of tissue homeostasis.^53–55,58^ At the end of the tissue regeneration process, the injury site is usually characterized by the presence of some level of fibrosis, which may vary based on the nature of the injury or the injury treatment.^33–35,53^ The metabolic activity of various aspects of this inflammatory response, such as the clearance of debris, immune infiltrate, and fibrotic tissue, may influence the signal, thus increasing the level of noise. Due to the presence of different cell types with different metabolic profiles, changes in the fluorescence lifetime within the muscle tissue before, during, and after contraction may be more difficult to detect.^28,58,59^

At the earlier timepoint, there was greater variability in the *A*_1_τ_1_/*A*_2_τ_2_ ratio between the three regions. The decrease in the *A*_1_τ_1_/*A*_2_τ_2_ ratio upon stimulation of contraction indicates a higher fraction of protein-bound NADH, suggesting an increase in the rate of ATP generation via OXPHOS.^2,28,49,50^ On day 14, there was very little variability in the *A*_1_τ_1_/*A*_2_τ_2_ ratio across the phases of contraction. Over time, inflammation decreases, and the tissue returns to a state of homeostasis, during which there is a metabolic shift from glycolysis to OXPHOS.^54^ This is mirrored in the *A*_1_τ_1_/*A*_2_τ_2_ ratios measured in healing muscle tissue suggesting that as the muscle tissue continues to heal, the mechanisms of energy generation begin to closely mimic that of uninjured muscle tissue.

Fractal dimension is a value that can describe how the time scale and the variance of a signal are related with higher fractal dimension values correlating to more complex signals.^52^ All fractal dimension values remained above the random walk threshold of 1.5. Trends showed that injured muscle tissue displayed lower fractal dimension values across the three regions of contraction, with greater variability between the regions. This indicates that the signals measured from injured muscle were less complex (lacking the highly nonstationary and nonlinear qualities associated with biological systems) than those of uninjured muscle tissue.^60,61^

The greatest variability in the fractal dimension was seen six days post-injury, with a decrease in variability 14 days post-injury. This suggests that as healing occurs, the fractal dimension likely begins to mimic that of uninjured tissue. This can be compared to other studies that have used fractal dimensions to measure changes in biological signals in response to various conditions, such as epileptic seizures, brain injury, and heart arrhythmias.^60,61^ A study investigating the changes in fractal dimension following injury of the parietal and cerebellar cortexes discovered that fractal dimension decreased immediately following injury and rose again with time. They discovered that this pattern continued with a repeated injury, showing lower FDs immediately following the injury.^61^ Future work using TCSPC measurements of NADH fluorescence will be required to examine the relationship between FD and injury, inflammation, and repair in skeletal muscle.

## 5. Conclusions

Overall, the results of this study provide sufficient evidence to suggest that fiber-based TCSPC can be used to measure NADH binding within muscle tissue. While there are some limitations with this method, such as a lack of spatial information, and no information about cellular structure or coenzyme concentration resulting in generalizations about the metabolic state, it can successfully measure NADH *in vivo* in a minimally invasive and nondestructive manner. Future studies are needed to further correlate the results of fiber-based TCSPC with well-established information by combining this technique with current standards. Fiber-based TCSPC has a large potential to advance research in areas from metabolic diseases, metabolic changes in inflammatory response, and muscular physiology by opening the doors to further studies *in vivo*.

This study had several limitations including small sample sizes for the uninjured (*n* = 2 for each timepoint) and injured (*n* = 2 for each timepoint) groups. Future studies with larger sample sizes are necessary to rigorously establish the significance of the preliminary data presented here. Another limitation of this study is the lack of more extensive validation testing, such as muscle proteomics, immunostaining, or collagen staining. Additionally, there are sources of error that are important to note. While contralateral controls serve as a standard method in VML studies, it is important to note that there is an increase in energy consumption within an animal during wound healing, which could result in systemic metabolic changes seen throughout the animal.^33–36^ Thus, it is important to note this as a potential source of error that could be minimized in future studies through the use of an external control model, such as uninjured animals and injured animals. In this study, all injuries were in the left TA muscle, but future studies will include randomly chosen injury site (left TA or right TA). There are also potential sources of error in the fiber-coupled TCSPC measurements, such as signal pollution from hemodynamic oscillations, the immune cells present in the tissue at each of the injury time points, or measurements subject to excess noise. Hemodynamic oscillations could have an effect on the measurements, for example, the heart rate of rats has been reported to have a frequency of 6 0:9 Hz, overlapping with the sampling frequency used (5 Hz).^62^ This could also serve as a potential source of error as signal aliasing could occur. Signal aliasing occurs when the sampling frequency for a signal is lower than the Nyquist rate, leading to overlapping frequency components.^63^ There are many biomedical signals that are limited by risks of aliasing, such as electrocardiography, electroencephalography, and magnetic resonance imaging.^64–66^ There are many factors that can affect the measured fluorescence of NADH including microenvironmental conditions, such as pH, polarity, etc., which are perturbed in muscle injury. Therefore, it is important to note that NADH quantum yield is a potential source of error.^67,68^ Finally, increasing the integration time of the data collection system should be considered in future studies to further minimize the risk of signal pollution due to noise.

Measurement of the level of specificity of this TCSPC system and further comparison to the gold standard methods of measuring the metabolic profiles of the tissue would aid in the improvement of future studies. This technique could be useful in future research and clinically to measure NADH binding, which could provide information on how NADH binding is affected by various conditions or diseases.

Fiber-coupled TCSPC has the potential to become an important resource in future research by allowing researchers to investigate NADH binding to a greater depth as a target for therapeutic monitoring and diagnostics. Further research consisting of much larger sets of data should be completed to help minimize measurement variation, to solidify the results presented in this paper, and to measure changes in NADH binding in other metabolic conditions. This method allows these measurements to be collected in an environment more comparable to clinical needs (i.e., animal model versus cell model). More research is needed to fully quantify the measurable range and sensitivity of this technique in living tissue and in an array of conditions such as cancer, injury, and metabolic diseases. Furthermore, fiber-coupled TCSPC has the potential to, one day, serve as a medical device used by clinicians for diagnostics and therapeutic monitoring of treatment and disease progression.

## Supplementary Material

Supplemental Material
