# Supplementary material for: In vivo measurement of NADH fluorescence lifetime in skeletal muscle via fiber-coupled time-correlated single photon counting
Source: J Innov Opt Health Sci. Author manuscript; Available in PMC 2025 Aug 28. (PMC12382436; doi:10.1142/s179354582350030x)
Supplement: Supplemental Material [file NIHMS2082360-supplement-Supplemental_Material.pdf]

Supplemental Information

Supplemental Table 1. Comparison of Fluorescence Lifetime Measurement Modalities[a]

	Fiber-coupled TCSPC	FLIM
**Spatial information**	None	Yes, image
**Data Acquisition**	Implanted needle, closed tissue	Surgical, open tissue
**Field Of View[b]**	10^8 μm^3	500 μm X 500 μm
**Depth limits**	Needle "optrode" can be inserted for measurements at depths beyond the tissue surface	Susceptible to scattering at surface of tissue, limited to depths up to a few hundred microns

[a]Fiber-coupled TCSPC is a point-based method of measuring fluorescence lifetime. Fluorescence lifetime imaging (FLIM) utilized the same TCSPC methodology in combination with microscopy. While FLIM provides spatial information of the fluorescence lifetime through microscopic images of the tissue/cells but has limited tissue depth capability and requires highly invasive techniques to provide access to the tissue of interest. Fiber-coupled TCSPC allows measurement of the same fluorescence lifetime at greater depths in vivo through the incorporation of a 26-gauge needle optrode that can be implanted into the tissue with minimal damage, but without the spatial information in the form of an image. [1-3]

[b]The field of view for fiber-coupled TCSPC is a volumetric average.

Supplemental Table 2. System Specifications and settings for data acquisition in the Becker & Hickl SPCM64 software[a]

**Operation Mode**	Continuous Flow Mode
**Collection Time**	0.2 s
**Laser Pulse Repetition Rate**	80 MHz
**Needle Size**	12mm, 26 Gauge
**Power at Sample**	20 μW
**Center Wavelength**	375 (+5/-10) nm
**Filter Parameters**	D = 22.4 mm, 450-700 nm AR coating
**CFD Limit High**	-50.98 mV
**CFD Limit Low**	-17.39 mV
**TAC Range**	5.003E-8 s
**TAC Gain**	4
**SYNC ZC Level**	-9.83 mV

[a]CFD: constant fraction discriminator; TAC: time to amplitude converter; ZC: zero crossing[2]

Supplemental Table 3. Settings used for NADH lifetime curve fitting in the Becker & Hickl SPCImage[a]

**Decay Type**	Multiexponential Decay
**Laser Repetition Time**	12.5 ns
**Laser Width**	50.0 ps
**Fit Method**	Maximum-likelihood estimation
**IRF Function**	$\frac{t}{80}e^{\frac{-t}{80}}$
**Position of IRF**	1.8 ns

[a]IRF: instrument response function

Supplemental Table 4. Ranges of R^2 values[a]

	Uninjured	Injured
**Before**	0.1136-0.7038	0.3521-0.3511
**During**	0.2613-0.8958	0.3455-0.7464
**After**	0.1407-0.5814	0.2968-0.8413

[a]Obtained for the calculation of the fractal dimension values for each group.

A comprehensive spreadsheet of the average A_1, A_2, τ_1, τ_2, Fractal Dimension, etc. for each animal, region of contraction, and timepoint can be found at the following location:

https://github.com/kmpriest/JIOHS-Publication/blob/main/NADH%20Lifetime%20and%20Fractal%20Dimension.xlsx

References

1. I. Georgakoudi and K. P. Quinn, "Optical imaging using endogenous contrast to assess metabolic state," Annu Rev Biomed Eng., **14**, 351-367 (2012).
2. M. Lukina, A. Orlova, M. Shirmanova, D. Shirokov, A. Pavlikov, A. Neubauer, H. Studier, W. Becker, E. Zagaynova, T. Yoshihara, S. Tobita, and V. Shcheslavskiy, "Interrogation of metabolic and oxygen states of tumors with fiber-based luminescence lifetime spectroscopy," Opt. Lett., **42(4)**, 731-734 (2017).
3. P. M. Schaefer, S. Kalinina, A. Rueck, C. A. F. von Arnim, and B. von Einem, "NADH autofluorescence: A marker on its way to boost bioenergetic research," Cytometry A, **95(1)**, 34-46 (2019).
